# Supplementary material for: Economic interests cloud hazard reductions in the European regulation of substances of very high concern
Source: Nat Commun. 2022 Nov 5;13:6686. doi: 10.1038/s41467-022-34492-2 (PMC9637112; doi:10.1038/s41467-022-34492-2)
Supplement: Supplementary file 1 — Supplementary Information [file 41467_2022_34492_MOESM1_ESM.docx]

**Supplementary Information**

**Economic Interests Cloud Hazard Reductions in the European Regulation of Substances of Very High Concern**

Jessica Coria^1^, Erik Kristiansson^2^ and Mikael Gustavsson^3^

(1) Department of Economics, University of Gothenburg, Gothenburg, Sweden. Corresponding Author. Email: [Jessica.Coria@economics.gu.se](mailto:Jessica.Coria@economics.gu.se)

(2) Department of Mathematical Sciences, Chalmers University of Technology/University of Gothenburg, Gothenburg, Sweden.

(3) Department of Economics, University of Gothenburg, Gothenburg, Sweden; and Department of Mathematical Sciences, Chalmers University of Technology/University of Gothenburg, Gothenburg, Sweden.

**Supplementary Table 1| Summary statistics.** Supplementary Table 1 summarizes the information collected for the compounds listed in either the REACH registry or the Candidate List.

|  | Variable | N | Mean | Std. Dev. | Min | Max |
| --- | --- | --- | --- | --- | --- | --- |
| Toxicological Properties | | | | | | |
| Carcinogenicity Warning | Fraction of notifiers that state the compound should be labelled as "Suspected of causing cancer" | 17093 | 0.018 | 0.122 | 0 | 1 |
| Carcinogenicity Danger | Fraction of notifiers that state the compound should be labelled as "May cause cancer" | 17093 | 0.029 | 0.157 | 0 | 1 |
| Mutagenicity Warning | Fraction of notifiers that state the compound should be labelled as "Suspected of causing genetic defects" | 17093 | 0.028 | 0.152 | 0 | 1 |
| Mutagenicity  Danger | Fraction of notifiers that state the compound should be labelled as "May cause genetic defects" | 17093 | 0.012 | 0.100 | 0 | 1 |
| Reproduction Warning | Fraction of notifiers that state the compound should be labelled as "Suspected of damaging fertility or the unborn child" | 17093 | 0.025 | 0.140 | 0 | 1 |
| Reproduction Danger | Fraction of notifiers that state the compound should be labelled as "May damage fertility or the unborn child" | 17093 | 0.022 | 0.139 | 0 | 1 |
| CMR Score | CMR score with range 0-1 | 17093 | 0.031 | 0.113 | 0 | 1 |
| Acute Environmental Warning | Fraction of notifiers that state the compound should be labelled as "Very toxic to aquatic life" | 17093 | 0.091 | 0.268 | 0 | 1 |
| Chronic Environmental Warning | Fraction of notifiers that state the compound should be labelled as "Very toxic to aquatic life with long lasting effects" | 17093 | 0.081 | 0.254 | 0 | 1 |
| Environmental Score | Environmental score with range 0-1 | 17093 | 0.086 | 0.248 | 0 | 1 |
| Economic Parameters | | | | | | |
| Tonnage | Tonnage band | 15169 | 1.564 | 1.699 | 0 | 9 |
| Countries | Number of countries in the European Economic Area with REACH registrants | 15169 | 1.927 | 2.574 | 0 | 30 |
| Knowledge Parameters | | | | | | |
| Publication Rank | Rank based on number publications. | 15169 | 0.227 | 0.593 | 0 | 4 |
| Publications | The total number of publications before implementation of CL. | 15169 | 8.362 | 115.877 | 0 | 7641 |

**Supplementary Method 1| List of Journals.** List of the 36 selected international peer-reviewed toxicological and ecotoxicological journals utilized to construct our proxy for scientific knowledge available

ARCHIVES OF ENVIRONMENTAL CONTAMINATION AND TOXICOLOGY

ARCHIVES OF TOXICOLOGY

AQUATIC TOXICOLOGY (AMSTERDAM, NETHERLANDS)

BULLETIN OF ENVIRONMENTAL CONTAMINATION AND TOXICOLOGY

CELL BIOLOGY AND TOXICOLOGY

CHEMICAL RESEARCH IN TOXICOLOGY

CHEMOSPHERE

CLINICAL TOXICOLOGY (PHILADELPHIA, PA.)

ECOTOXICOLOGY (LONDON, ENGLAND)

ECOTOXICOLOGY AND ENVIRONMENTAL SAFETY

ENVIRONMENTAL HEALTH PERSPECTIVES

ENVIRONMENT INTERNATIONAL

ENVIRONMENTAL POLLUTION (BARKING, ESSEX: 1987)

ENVIRONMENTAL SCIENCE AND POLLUTION RESEARCH INTERNATIONAL

ENVIRONMENTAL TOXICOLOGY AND PHARMACOLOGY

ENVIRONMENTAL TOXICOLOGY

ENVIRONMENTAL TOXICOLOGY AND CHEMISTRY

FOOD AND CHEMICAL TOXICOLOGY: AN INTERNATIONAL JOURNAL PUBLISHED FOR THE BRITISH

HUMAN &AMP; EXPERIMENTAL TOXICOLOGY

INDUSTRIAL BIOLOGICAL RESEARCH ASSOCIATION

INHALATION TOXICOLOGY

JOURNAL OF ANALYTICAL TOXICOLOGY

JOURNAL OF APPLIED TOXICOLOGY: JAT

MUTAGENESIS

MUTATION RESEARCH. GENETIC TOXICOLOGY AND ENVIRONMENTAL MUTAGENESIS

NANOTOXICOLOGY

NEUROTOXICOLOGY AND TERATOLOGY

NEUROTOXICITY RESEARCH

REPRODUCTIVE TOXICOLOGY (ELMSFORD, N.Y.)

THE SCIENCE OF THE TOTAL ENVIRONMENT

TOXICOLOGY

TOXICOLOGY LETTERS

TOXICOLOGIC PATHOLOGY

TOXICOLOGICAL AND ENVIRONMENTAL CHEMISTRY

TOXICOLOGICAL SCIENCES

TOXICOLOGICAL SCIENCES: AN OFFICIAL JOURNAL OF THE SOCIETY OF TOXICOLOGY

**Logistic Regressions Candidate List**

**Supplementary Table 2| Logistic Regressions with CMR and Environmental Scores as proxies for toxicological properties.** The parameter estimates and p-values from the logistic regression models where the likelihood of inclusion on the Candidate List is predicted by the toxicological properties of the substances as well as the economic and knowledge parameters. Chemicals listed in the Candidate List are compared to all chemicals registered under the European chemical regulation Registration, Evaluation, Authorization, and Restriction of Chemicals (REACH), to chemicals listed on the SIN list (SIN), and to chemicals listed on the PRIO list (PRIO). The CMR Score measures Carcinogenicity (C), Mutagenicity (M), and Reproductive toxicity (R), and the Environmental Score measures environmental hazard. In columns (1), (4) and (7), the probability of inclusion on the Candidate List is explained only as a function of the CMR Score and Environmental Score. In columns (2), (5) and (8), we added proxies for economic motivations, and, in columns (3), (6) and (9), we also added the scientific knowledge available (measured by the Publication Rank). The model fit is reported as the McFadden adjusted R^2^.

|  |  | REACH |  |  | SIN |  |  | PRIO |  |
| --- | --- | --- | --- | --- | --- | --- | --- | --- | --- |
|  | (1) | (2) | (3) | (4) | (5) | (6) | (7) | (8) | (9) |
| CMR Score | 4.84*** (6.1E-91) | 5.02*** (5.7E-82) | 5.21*** (2.4E-80) | -1.72*** (3.8E-08) | -1.63*** (6.2E-07) | -1.44*** (1.3E-05) | -0.04 (8.9E-01) | -0.56 (1.1E-01) | -0.55 (1.1E-01) |
| Environmental Score | 1.84*** (1.4E-29) | 2.07*** (6.9E-34) | 1.86*** (1.3E-25) | 0.84*** (3.4E-06) | 0.70*** (2.7E-04) | 0.68*** (4.1E-04) | -0.67*** (6.5E-05) | -0.53** (4.1E-03) | -0.50** (7.5E-03) |
| Tonnage Band |  | 1.05** (7.9E-03) | 1.37*** (8.5E-04) |  | -3.24*** (2.7E-08) | -2.88*** (1.4E-06) |  | 2.63** (1.2E-03) | 2.66** (1.1E-03) |
| Sqrt(#Countries) |  | -4.85*** (1.9E-13) | -6.25*** (6.9E-21) |  | 3.13*** (4.2E-06) | 2.65*** (1.5E-04) |  | 1.25 (1.3E-01) | 1.03 (2.2E-01) |
| Publication Rank |  |  | 3.83*** (2.7E-45) |  |  | 0.92** (1.9E-03) |  |  | 0.68** (2.9E-02) |
|  |  |  |  |  |  |  |  |  |  |
| # Obs | 17093 | 15169 | 15169 | 910 | 874 | 874 | 892 | 856 | 856 |
| R^2^ | 0.21 | 0.24 | 0.31 | 0.04 | 0.06 | 0.07 | 0.01 | 0.1 | 0.1 |

Notes: Estimated coefficients of logistics regressions. Standard errors in parentheses.

*** Significant at 0.01, **Significant at 0.05, *Significant at 0.1

**Supplementary Table 3| Logistic Regressions with more detailed toxicological information.** The parameter estimates and p-values from the logistic regression models where we explore in further detail which specific toxicological properties affect the listing on the Candidate List. Chemicals listed in the Candidate List are compared to all chemicals registered under the European chemical regulation Registration, Evaluation, Authorization, and Restriction of Chemicals (REACH), to chemicals listed on the SIN list (SIN), and to chemicals listed on the PRIO list (PRIO). In columns (1), (4) and (7), the probability of inclusion on the Candidate List is explained only as a function of the toxicological variables (measured by Carcinogenicity, Mutagenicity, Reproductive Toxicity warning and danger and acute and chronic environmental warnings). In columns (2), (5) and (8), we added proxies for economic motivations. In columns (3), (6) and (9), we also added the scientific knowledge available. The model fit is reported as the McFadden adjusted R^2^.

|  |  | REACH |  |  | SIN |  |  | PRIO |  |
| --- | --- | --- | --- | --- | --- | --- | --- | --- | --- |
|  | (1) | (2) | (3) | (4) | (5) | (6) | (7) | (8) | (9) |
| Carcinogenicity Warning | 0.41 (2.3E-01) | 0.60* (7.6E-02) | 0.02 (9.6E-01) | -0.61 (1.1E-01) | -0.54 (1.8E-01) | -0.62 (1.2E-01) | 0.01 (9.7E-01) | 0.02 (9.5E-01) | -0.09 (8.2E-01) |
| Carcinogenicity Danger | 1.74*** (5.5E-12) | 1.92*** (2.3E-13) | 1.92*** (1.1E-12) | -1.78*** (6.7E-14) | -1.88*** (6.8E-14) | -1.77*** (2.7E-12) | -0.93*** (2.6E-05) | -1.16*** (2.3E-06) | -1.15*** (2.7E-06) |
| Mutagenicity Warning | -0.82** (2.1E-02) | -0.60* (9.0E-02) | -0.55 (1.4E-01) | -0.50 (1.4E-01) | -0.57 (1.0E-01) | -0.59* (9.2E-02) | -0.24 (4.7E-01) | -0.26 (4.6E-01) | -0.29 (4.1E-01) |
| Mutagenicity Danger | -0.35 (3.5E-01) | -0.61 (1.1E-01) | -0.54 (2.0E-01) | -0.06 (8.6E-01) | 0.40 (2.8E-01) | 0.37 (3.3E-01) | 1.14** (1.6E-03) | 1.36*** (2.0E-03) | 1.25*** (5.1E-03) |
| Reproduction Warning | 2.04*** (8.4E-16) | 2.03*** (1.1E-14) | 2.04*** (7.3E-14) | 0.80** (1.7E-02) | 1.09** (2.1E-03) | 1.11** (1.6E-03) | 1.93** (2.9E-03) | 1.98*** (9.9E-08) | 1.98** (1.1E-07) |
| Reproduction Danger | 3.25*** (2.9E-64) | 3.24*** (1.3E-60) | 3.37*** (5.4E-61) | 0.89*** (5.3E-05) | 0.88*** (1.4E-04) | 0.97*** (3.4E-05) | 0.57** (2.9E-03) | 0.34 (1.1E-01) | 0.38* (6.9E-02) |
| Acute Environ. Warning | 0.88** (2.7E-03) | 0.96*** (9.4E-04) | 0.74** (1.6E-02) | 0.89* (6.8E-02) | 0.89* (8.4E-02) | 0.78 (1.3E-01) | 0.64 (1.1E-01) | 0.86** (4.6E-02) | 0.82* (5.9E-02) |
| Chronic Environ. Warning | 0.69** (2.0E-02) | 0.81** (6.2E-03) | 0.91** (3.9E-03) | -0.05 (9.3E-01) | -0.09 (8.5E-01) | -0.03 (9.6E-01) | -1.35*** (6.8E-04) | -1.46*** (6.4E-04) | -1.40** (1.2E-03) |
| Tonnage Band |  | 1.64*** (1.1E-04) | 2.03*** (5.3E-06) |  | -3.45*** (1.2E-07) | -3.11*** (3.3E-06) |  | 2.91*** (7.0E-04) | 2.90*** (7.3E-04) |
| sqrt(#Countries) |  | -5.21*** (1.4E-14) | -6.55*** (2.7E-21) |  | 3.03*** (4.6E-05) | 2.57*** (7.8E-04) |  | 0.88 (3.2E-01) | 0.73 (4.1E-01) |
| Publication Ranking |  |  | 3.90*** (2.7E-43) |  |  | 0.85** (8.4E-03) |  |  | 0.59* (7.7E-02) |
|  |  |  |  |  |  |  |  |  |  |
| # Obs | 17093 | 15169 | 15169 | 910 | 874 | 874 | 892 | 856 | 856 |
| R^2^ | 0.25 | 0.27 | 0.34 | 0.12 | 0.16 | 0.16 | 0.07 | 0.16 | 0.16 |

Notes: Notes: Estimated coefficients of logistics regressions. Standard errors in parentheses.

*** Significant at 0.01, **Significant at 0.05, *Significant at 0.1

**Logistic Regressions Authorization List**

**Supplementary Table 4| Logistic Regressions with CMR and Environmental Scores as proxies for toxicological properties.** The parameter estimates and p-values from the logistic regression models where the likelihood of inclusion on the Authorization List is predicted by the toxicological properties of the substances as well as the economic and knowledge parameters. Chemicals listed in the Authorization List are compared to all chemicals registered under the European chemical regulation Registration, Evaluation, Authorization, and Restriction of Chemicals (REACH), to chemicals listed on the SIN list (SIN), and to chemicals listed on the PRIO list (PRIO). The CMR Score measures Carcinogenicity (C), Mutagenicity (M), and Reproductive toxicity (R), and the Environmental Score measures environmental hazard. In columns (1), (4) and (7), the probability of inclusion on the Authorization List is explained only as a function of the CMR Score and Environmental Score. In columns (2), (5) and (8), we added proxies for economic motivations, and, in columns (3), (6) and (9), we also added the scientific knowledge available (measured by the Publication Rank). The model fit is reported as the McFadden adjusted R^2^.

|  |  | REACH |  |  | SIN |  |  | PRIO |  |
| --- | --- | --- | --- | --- | --- | --- | --- | --- | --- |
|  | (1) | (2) | (3) | (4) | (5) | (6) | (7) | (8) | (9) |
| CMR Score | 4.27*** (7.1E-30) | 4.8*** (1.5E-32) | 4.91*** (1.6E-31) | -1.51*** (1.9E-06) | -1.41*** (2.1E-05) | -1.22*** (2.6E-04) | 0.1 (7.4E-01) | -0.46 (2.0E-01) | -0.46 (2.0E-01) |
| Environmental Score | 1.86*** (1.4E-11) | 2.24*** (2.6E-15) | 1.98*** (1.6E-11) | 0.77*** (2.7E-05) | 0.63*** (1.2E-03) | 0.61*** (1.8E-03) | -0.69*** (6.3E-05) | -0.54*** (5.7E-03) | -0.49*** (1.2E-02) |
| Tonnage Band |  | 1.27* (5.0E-02) | 1.53** (2.4E-02) |  | -3.34*** (1.3E-08) | -2.99*** (7.0E-07) |  | 2.7*** (1.2E-03) | 2.75*** (1.1E-03) |
| Sqrt(#Countries) |  | -9.23*** (1.1E-14) | -10.2*** (1.4E-17) |  | 3.35*** (1.1E-06) | 2.87*** (5.1E-05) |  | 1.39 (1.0E-01) | 1.12 (1.9E-01) |
| Publication Rank |  |  | 3.37*** (1.9E-13) |  |  | 0.94*** (1.6E-03) |  |  | 0. 85*** (7.8E-03) |
|  |  |  |  |  |  |  |  |  |  |
| # Obs | 16925 | 15006 | 15006 | 897 | 861 | 861 | 873 | 837 | 837 |
| R^2^ | 0.16 | 0.23 | 0.28 | 0.03 | 0.06 | 0.07 | 0.01 | 0.11 | 0.12 |

Notes: Estimated coefficients of logistics regressions. Standard errors in parentheses.

*** Significant at 0.01, **Significant at 0.05, *Significant at 0.1

The odd ratios of the coefficients in cols (3), (6) and (9) are presented graphically in Supplementary Figure 1.


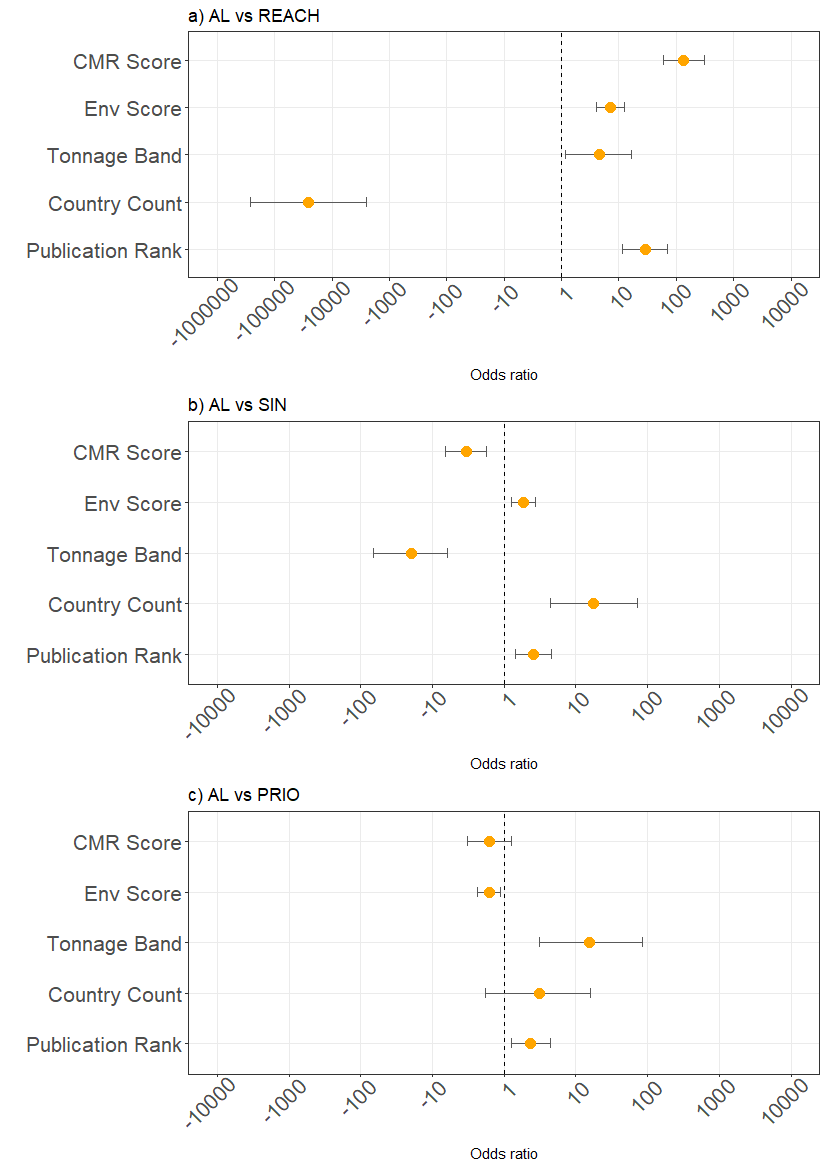


**Supplementary Figure 1| Marginal Contribution of Explanatory Variables to the Odds of Inclusion on Authorization List.** The figure presents the point estimates of the odds-ratio and the corresponding 95% confidence interval for all parameters included in the logit model. Chemicals listed in the Authorization List (AL) are compared to a) all chemicals registered under the European chemical regulation Registration, Evaluation, Authorization, and Restriction of Chemicals (REACH, n=15,006), b) chemicals listed on the SIN list (SIN, n = 861), c) chemicals listed on the PRIO list (PRIO, n = 837). Parameters with estimates to the left of the vertical line at 1 will typically be of lower value in the AL as compared to the list with which it is compared, and vice versa.

**Supplementary Table 5| Logistic Regressions with more detailed toxicological information.** The parameter estimates and p-values from the logistic regression models where we explore in further detail which specific toxicological properties affect the listing on the Authorization List. Chemicals listed in the Authorization List are compared to all chemicals registered under the European chemical regulation Registration, Evaluation, Authorization, and Restriction of Chemicals (REACH), to chemicals listed on the SIN list (SIN), and to chemicals listed on the PRIO list (PRIO). In columns (1), (4) and (7), the probability of inclusion on the Authorization List is explained only as a function of the toxicological variables (measured by Carcinogenicity, Mutagenicity, Reproductive Toxicity warning and danger and acute and chronic environmental warnings). In columns (2), (5) and (8), we added proxies for economic motivations. In columns (3), (6) and (9), we also added the scientific knowledge available. The model fit is reported as the McFadden adjusted R^2^.

|  |  | REACH |  |  | SIN |  |  | PRIO |  |
| --- | --- | --- | --- | --- | --- | --- | --- | --- | --- |
|  | (1) | (2) | (3) | (4) | (5) | (6) | (7) | (8) | (9) |
| Carcinogenicity Warning | -0.03 (9.7E-01) | 0.03 (9.6E-01) | -0.64 (3.9E-01) | -0.52 (1.7E-01) | -0.45 (2.6E-01) | -0.54 (1.8E-01) | 0.02 (9.6E-01) | 0.00 (10.0E-01) | -0.16 (6.9E-01) |
| Carcinogenicity Danger | 2.09*** (6.8E-8) | 2.78*** (1.2E-11) | 2.93*** (3.3E-13) | -1.65*** (5.2E-12) | -1.75*** (4.5E-12) | -1.64*** (1.3E-10) | -0.83*** (2.1E-04) | -1.05*** (2.3E-05) | -1.04*** (2.8E-05) |
| Mutagenicity Warning | -1.67*** (5.4E-03) | -1.85*** (4.8E-03) | -1.82*** (8.4E-03) | -0.52 (1.3E-01) | -0.59* (9.4E-02) | -0.60* (8.5E-02) | -0.21 (5.1E-01) | -0.24 (5.0E-01) | -0.28 (4.4E-01) |
| Mutagenicity Danger | -0.57 (3.1E-01) | -0.86 (1.4E-01) | -0.99* (9.7E-02) | -0.04 (9.1E-01) | 0.40 (2.9E-01) | 0.37 (3.3E-01) | 0.94** (1.2E-02) | 0.98** (3.4E-02) | 0.79* (9.2E-02) |
| Reproduction Warning | 1.43*** (4.8E-3) | 1.31** (1.3E-02) | 1.24** (2.5E-02) | 0.71** (4.6E-02) | 0.99*** (7.3E-03) | 0.99*** (7.1E-03) | 1.77*** (1.1E-06) | 1.75*** (1.1E-05) | 1.73*** (1.5E-05) |
| Reproduction Danger | 2.76*** (1.3E-15) | 2.85*** (8.8E-18) | 2.83*** (1.5E-16) | 0.96*** (1.3E-05) | 0.95*** (3.9E-05) | 1.04*** (9.6E-06) | 0.67*** (5.3E-04) | 0.45** (3.6E-02) | 0.52** (1.6E-02) |
| Acute Environ. Warning | 1.54*** (3.7E-04) | 1.53*** (3.2E-04) | 1.44*** (1.2E-03) | 0.93* (5.8E-02) | 0.95* (6.5E-02) | 0.84 (1.0E-01) | 0.57 (1.6E-01) | 0.8* (7.0E-02) | 0.74* (9.7E-02) |
| Chronic Environ. Warning | 0.11 (8.0E-01) | 0.52 (2.4E-01) | 0.41 (3.9E-01) | -0.15 (7.6E-01) | -0.21 (6.9E-01) | -0.14 (7.8E-01) | -1.3*** (1.1E-03) | -1.39*** (1.4E-03) | -1.3*** (3.1E-03) |
| Tonnage Band |  | 1.38** (4.8E-02) | 1.77** (1.5E-02) |  | -3.53*** (8.7E-08) | -3.2*** (2.2E-06) |  | 2.79*** (1.4E-03) | 2.80*** (1.4E-03) |
| sqrt(#Countries) |  | -9.6*** (1.3E-15) | -10.5*** (3.9E-18) |  | 3.24*** (1.5E-05) | 2.80*** (2.9E-04) |  | 1.16 (2.0E-01) | 0.95 (2.9E-01) |
| Publication Ranking |  |  | 3.44*** (5.1E-13) |  |  | 0.85*** (9.1E-03) |  |  | 0.81** (1.8E-02) |
|  |  |  |  |  |  |  |  |  |  |
| # Obs | 16925 | 15006 | 15006 | 897 | 861 | 861 | 873 | 837 | 837 |
| R^2^ | 0.19 | 0.26 | 0.30 | 0.11 | 0.15 | 0.15 | 0.06 | 0.16 | 0.16 |

Notes: Notes: Estimated coefficients of logistics regressions. Standard errors in parentheses.

*** Significant at 0.01, **Significant at 0.05, *Significant at 0.1

The odd ratios of the coefficients in cols (3), (6) and (9) are presented graphically in Supplementary Figure 2.


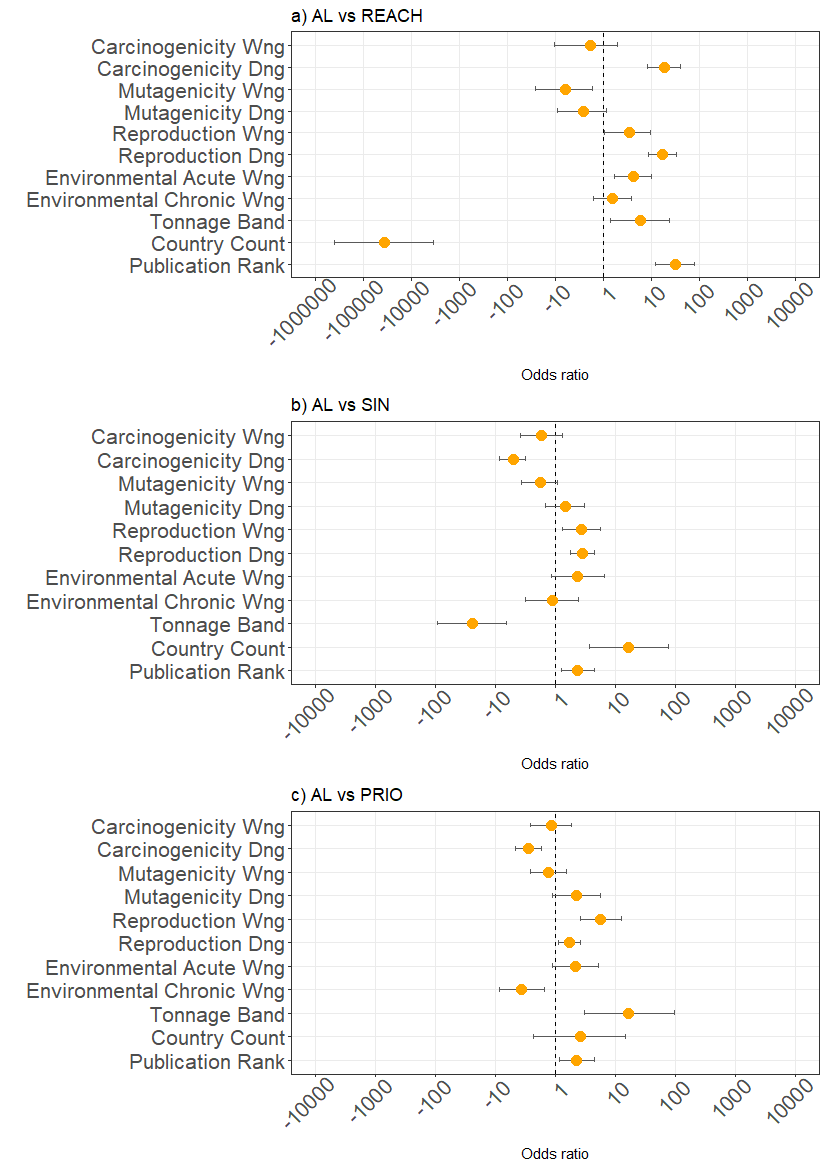


**Supplementary Figure 2| Marginal Contribution of Carcinogenicity, Mutagenicity, Reproductive Toxicity, and Environmental Properties to the Odds of Inclusion on the Authorization List.** The figure presents the point estimates of the odds-ratio and the corresponding 95% confidence interval for all parameters included in the logit model. Chemicals listed in the Authorization List (AL) are compared to a) all chemicals registered under the European chemical regulation Registration, Evaluation, Authorization, and Restriction of Chemicals (REACH, n = 15,006), b) chemicals listed on the SIN list (SIN, n = 861), c) chemicals listed on the PRIO list (PRIO, n = 837). Parameters with estimates to the left of the vertical line at 1 will typically be of lower value in the AL as compared to the list with which it is compared, and vice versa.

**Supplementary Information| Data and Code Repositories.**

This document contains a description of the input files and code in the supporting material repository. The code generates all data for the figures and tables in the manuscript and the supplementary information.

**File descriptions**

**File: “Analysis of SVHC data Candidate List.R”**

**The code generates all data reported in the corresponding article. Logit-modelling is performed comparing the compounds which are on the Candidate list to compounds on other lists.**

**File: “Analysis of SVHC data Authorization List.R”**

**Performs the same logit analysis as the code in the file “Analysis of SVHC data Candidate List.R”, but now comparing the compounds on the Authorization list to compounds on all other lists.**

**File: ”CLP Scoring.txt”**

CLP data was downloaded 2019-05-11. The data has been treated such that the value for each GHS code is the percentage of notifiers which have said that the particular compound should have the particular GHS code. The script aggregates the GHS codes in accordance with their signal words “Warning” or “Danger”. More information on GHS classification can be found at https://pubchem.ncbi.nlm.nih.gov/ghs/.

Note 1) For some individual compounds no notifiers were present, but the compound was still judged to have specific GHS codes associated with it due to harmonized classifications. For such compounds it was assumed that 100% of notifiers had claimed that the specific GHS codes should apply. Note 2) The CAS-numbers are not necessarily one per row in the input file. In this file I have refrained from expanding the data to multiple rows. Note 3) The pictogram assignment was only available for ~4600 chemicals.

Column details:

| Row_Number | Internal Reference number |
| --- | --- |
| Index_no. | ECHA index number |
| EC | EINECS-number |
| CAS_Number | CAS, including hyphen |
| CAS | CAS, excluding hyphen |
| Name | Name of compound |
| Page_ID | ECHA Page_ID |
| Link | ECHA Compound Link |
| Corrosion | Does the compound warrant the corresponding hazard pictogram |
| … | … |
| Skull_and_crossbones | Does the compound warrant the corresponding hazard pictogram |
| H200 | Percentage pf Notifiers assigning the respective GHS classification |
| … | … |
| Not_Classified | Percentage pf Notifiers assigning the respective GHS classification |
| Number of products_tot | Number of products as mentioned in the CLP |

**File: “Listing Information.txt”**

Information on which lists a compound could be found on, generated from all unique EC/CAS-numbers mentioned in the specific lists. It should be noted that to facilitate this study we have expanded the lists when more than one CAS or EC have been mentioned per listing. E.g., if the listing covered one EC and two CAS it was expanded to two lines. If the listing covered two EC and two CAS it was expanded to four lines. (i.e., all possible combinations). Expansions was comparatively rare, and it should have little to no effect on the end result.

For SVHC_AUTH the list was expanded once (Double CAS).

For SVHC_CAND the list was expanded five times (3-Double CAS, One Trippel CAS).

The PRIO_LIST had no expansions.

The SIN_LIST had 12 double CAS and Two Trippel CAS. It also had 7 Double EC and one 9-EC listing (Carbon nanotubes).

The REACH_REG had no expansions.

Column details:

| EC | EINECS number |
| --- | --- |
| IS_REACH_REGI | Is the compound registered under REACH |
| IS_SVHC_AUTH | Is the compound on the Authorization List |
| IS_SVHC_CAND | Is the compound on the Candidate List |
| IS_SIN_LIST | Is the compound in the SIN-list |
| IS_PRIO_PHASE_OUT | Is the compound on the PRIO list as phase out (used in publication) |
| IS_PRIO_PHASE_OUT_NOT_VALID | Is the compound on the PRIO list as phase out, but not always valid (there are exceptions for the marking included in classification if companies can show that certain components are present at low enough concentrations |
| IS_PRIO_PRIORITY | Is the compound on the PRIO list as a priority compound (lower urgency than phase out) |
| SVHC_CANDIDATE_INCLUSION_YEAR | Which year was the compound included on the Candidate list |

**File: “Publication Scores.txt”**

The publication frequency counts. I.e., the number of times the compound is mentioned in either of the publications included in the search.

Column details:

| EC | EINECS number |
| --- | --- |
| Lists | Compound listing information |
| Names | Cas and name of compound |
| Freq_Count_total_all | Total number of publications mentioning the compound |
| Counts[1966] | Number of publications per [year] |
| … | … |
| Counts[2020] | Number of publications per [year] |
| PMIDs[1966] | PubMedID for all publications mentioning the compound [year] |
| … | … |
| PMIDs[2020] | PubMedID for all publications mentioning the compound [year] |
| SVHC_CANDIDATE_INCLUSION_YEAR | Year the compound was included on the SVHC candidate list |
| Freq_Count_total_all_before_SVHC_candidate_inclusion | Number of publications before SVHC candidate list inclusion |
| SVHC_AUTHORIZATION_LATEST_APPLICATION_YEAR | Year the compound had last application year for Authorization |
| Freq_Count_total_all_before_SVHC_authorization_latest_application_date | Number of publications before SVHC authorization latest application date |
| SVHC_AUTHORIZATION_SUNSET_YEAR | Sunset year for compounds on Authorization list |
| Freq_Count_total_all_before_SVHC_authorization_sunset_date | Number of publications before SVHC authorization sunset date |

**File: “REACH Substance Export 20200225.txt”**

The tonnage information is a direct download from the REACH registry. The download was performed 2020-02-25. The data has been treated and the tonnage band shifted in accordance with the scheme below.

Note 1) Tonnages get sorted and the duplicates are removed. This ensures that only the highest Tonnage_Band per unique EC is kept.

Tonnage_Band

Confidential = NA

Intermediate = 0

1-10 = 2

10-100 = 3

100-1000 = 4

1000-10000 = 5

10000-100000 = 6

100000-1000000 = 7

1000000-10000000 = 8

10000000-100000000 = 9

Column details:

| Name | Name of compound |
| --- | --- |
| EC | EINECS-number |
| Cas Number | CAS-number |
| Registration Type | Which type of registration has been done under REACH |
| Submission Type | Type of submission to ECHA |
| Tonnage_Band | Log-transformed Tonnage band (import+production) the compound belongs to |
| Total tonnage Band | Tonnage_band as mentioned in the downloaded registry file |
| Factsheet URL | Direct link to ECHA factsheet |
| Substance Information Page | Direct link to ECHA substance information page |

**File: “Reader Registrations 20190725.txt”**

Reach registration data was gathered 2019-07-11. For an example of the web-pages the data was collected from see for example <https://echa.europa.eu/sv/registration-dossier/-/registered-dossier/15858>/1/2.

The data is treated to aggregate to

1) The sum of all registrants per EC.

2) The number of inactive and active registrants per country.

3) The total number of registrants per EC (Active + Inactive)

4) The percentage of Inactive registration numbers

5) The total number of countries which have an active or inactive registrant

Column details:

| Row | Internal indexation number |
| --- | --- |
| CAS | CAS-number |
| EC | EINECS-number |
| ActiveRegistrants | Total number of active registrants as listed under “Registrants/Suppliers -Active.” |
| InActiveRegistrants | Total number of active registrants as listed under “Registrants/Suppliers -Inactive. |
| ActiveRegNumbers | Total number of registration numbers under heading “Registration numbers - Active” |
| InActiveRegNumbers | Total number of registration numbers under heading “Registration numbers - Inactive 1 |
| ActiveAustria | Total number of active registrants from [Country] |
| … | … |
| ActiveConfidential | Total number of active registrants from [Country] |
| InActiveAustria | Total number of inactive registrants from [Country] |
| … | … |
| InActiveConfidential | Total number of inactive registrants from [Country] |
