# Supplementary material for: Economic interests cloud hazard reductions in the European regulation of substances of very high concern
Source: Nat Commun. 2022 Nov 5;13:6686. doi: 10.1038/s41467-022-34492-2 (PMC9637112; doi:10.1038/s41467-022-34492-2)
Supplement: Supplementary file 3 — Reporting Summary [file 41467_2022_34492_MOESM3_ESM.pdf]

Corresponding author(s): CORIA, JESSICA

Last updated by author(s): Okt 14, 2022

Reporting Summary

Nature Portfolio wishes to improve the reproducibility of the work that we publish. This form provides structure for consistency and transparency in reporting. For further information on Nature Portfolio policies, see our Editorial Policies and the Editorial Policy Checklist.

Statistics

For all statistical analyses, confirm that the following items are present in the figure legend, table legend, main text, or Methods section.

n/a	Confirmed	
☐	☒	The exact sample size (*n*) for each experimental group/condition, given as a discrete number and unit of measurement
☐	☒	A statement on whether measurements were taken from distinct samples or whether the same sample was measured repeatedly
☐	☒	The statistical test(s) used AND whether they are one- or two-sided<br>*Only common tests should be described solely by name; describe more complex techniques in the Methods section.*
☐	☒	A description of all covariates tested
☐	☒	A description of any assumptions or corrections, such as tests of normality and adjustment for multiple comparisons
☐	☒	A full description of the statistical parameters including central tendency (e.g. means) or other basic estimates (e.g. regression coefficient) AND variation (e.g. standard deviation) or associated estimates of uncertainty (e.g. confidence intervals)
☐	☒	For null hypothesis testing, the test statistic (e.g. *F*, *t*, *r*) with confidence intervals, effect sizes, degrees of freedom and *P* value noted<br>*Give P values as exact values whenever suitable.*
☒	☐	For Bayesian analysis, information on the choice of priors and Markov chain Monte Carlo settings
☒	☐	For hierarchical and complex designs, identification of the appropriate level for tests and full reporting of outcomes
☒	☐	Estimates of effect sizes (e.g. Cohen's *d*, Pearson's *r*), indicating how they were calculated

Our web collection on statistics for biologists contains articles on many of the points above.

Software and code

Policy information about availability of computer code

Data collection	Data-gathering from the Classification and Labeling Inventory and the REACH dossiers was performed using R version 3.6.1 and a standard XML library.<br><br>All other datasets were downloaded directly from their respective sources (see separate Data Collection heading below).
Data analysis	Apart from the PubMed analysis and the calculation of percentages for all Classification and Labelling entries all analysis was performed using R version 4.1. The following libraries were employed during the course of data-treatment and visualization: ggplot2 v3.3.5, tidyverse v1.3.1 and stringr v1.4.0.<br><br>Initail processing (i.e. counting number of publications) of the PubMed-data was performed using Python and a standard XML-library.<br><br>Initial processing (i.e. calculating percentages for each hazard-code) of the Classification and Labelling data was done using VBA.<br><br>All code used to analyze the the data in the study are available in an online repository with DOI:10.5281/zenodo.7051114

For manuscripts utilizing custom algorithms or software that are central to the research but not yet described in published literature, software must be made available to editors and reviewers. We strongly encourage code deposition in a community repository (e.g. GitHub). See the Nature Portfolio guidelines for submitting code & software for further information.

Data

Policy information about availability of data

All manuscripts must include a data availability statement. This statement should provide the following information, where applicable:

- Accession codes, unique identifiers, or web links for publicly available datasets
- A description of any restrictions on data availability
- For clinical datasets or third party data, please ensure that the statement adheres to our policy

The data and code generated and analyzed during the current study are available in the Zenodo data repository; Data for " Economic Interests Cloud Hazard Reductions in the European Regulation of Substances of Very High Concern"”; Doi: 10.5281/zenodo.7051114.

Human research participants

Policy information about studies involving human research participants and Sex and Gender in Research.

Reporting on sex and gender	*Use the terms sex (biological attribute) and gender (shaped by social and cultural circumstances) carefully in order to avoid confusing both terms. Indicate if findings apply to only one sex or gender; describe whether sex and gender were considered in study design whether sex and/or gender was determined based on self-reporting or assigned and methods used. Provide in the source data disaggregated sex and gender data where this information has been collected, and consent has been obtained for sharing of individual-level data; provide overall numbers in this Reporting Summary. Please state if this information has not been collected. Report sex- and gender-based analyses where performed, justify reasons for lack of sex- and gender-based analysis.*
Population characteristics	*Describe the covariate-relevant population characteristics of the human research participants (e.g. age, genotypic information, past and current diagnosis and treatment categories). If you filled out the behavioural & social sciences study design questions and have nothing to add here, write "See above."*
Recruitment	*Describe how participants were recruited. Outline any potential self-selection bias or other biases that may be present and how these are likely to impact results.*
Ethics oversight	*Identify the organization(s) that approved the study protocol.*

Note that full information on the approval of the study protocol must also be provided in the manuscript.

Field-specific reporting

Please select the one below that is the best fit for your research. If you are not sure, read the appropriate sections before making your selection.

☐ Life sciences ☐ Behavioural & social sciences ☒ Ecological, evolutionary & environmental sciences

For a reference copy of the document with all sections, see nature.com/documents/nr-reporting-summary-flat.pdf

Ecological, evolutionary & environmental sciences study design

All studies must disclose on these points even when the disclosure is negative.

Study description	In this paper, we investigate what drives the regulation of substances of very high concern in Europe. The Candidate List was established as a part of the European regulation on the Registration, Evaluation, Authorization and Restriction of Chemicals (REACH) to encourage manufacturers to remove substances of very high concern from the market. We constructed a unique dataset that allowed us to characterize how hazardousness, economic interests, and available scientific information have shaped the Candidate List. Our dataset is based on secondary sources of information available in open sources online. We made use of standard statistical tests and logistic regression to analyze the relative importance of toxicological, economic and and knowledge parameters on the probability of inclusion on the Candidate List.
Research sample	In addition to comparing the chemicals on the Candidate List to all compounds registered under REACH we also compare the Candidate list chemicals to two alternative samples of chemicals. First, the SIN list of hazardous substances developed by the International Chemical Secretariat, ChemSec (an independent non-profit organization that advocates for substitution of toxic chemicals). Second, a list of hazardous substances that should be phased out, developed by the Swedish Chemical Agency (PRIO-list).<br><br>We used logistic regressions to analyze the relative importance of toxicological, economics, and available scientific knowledge on the probability of inclusion on the Candidate List, and the differences between the Candidate List and REACH, SIN, and PRIO. We also verified whether our findings hold in the analysis of the probability of inclusion on the Authorization List (which corresponds to the regulatory list including a subset of substances on the Candidate List subjected to binding restrictions).<br><br>To assess the importance of the drivers of inclusion on the Candidate List data on each compounds hazardousness, economic

importance and scientific knowledge was needed.

Data on the hazardousness of substances was collected from the European Chemicals Agency (ECHA) Classification and Labelling Inventory. The hazardousness of a compound was determined per GHS code as the percentage of notifiers registering the compound as having that GHS code.

Data on the economic variables ("total tonnage" and "number of producing/importing countries") was gathered from REACH registration dossiers, kept by ECHA.

Data on number of studies performed per individual compound was gathered from PubMed, as held by the National Centre for Biotechnology Information (NCBI).

Lists of compounds belonging the the Candidate List and the Authorization List was gathered from ECHAs official website, while the SIN and PRIO list was gathered from the hompages of the International Chemical Secretariat (ChemSec) and the Swedish Chemicals Agency (KEMI), respectively.

Since the Candidate List include substances that may have serious effects on human health and the environment, we expected the relative importance of hazardousness to be more salient when comparing the Candidate List to the bulk of substances registered under REACH. Since SIN and PRIO are focused on the listing of hazardous substances, we expected the relative importance of economic interests and knowledge to be more salient when comparing the Candidate List to them.

Sampling strategy

No sample size calculations were performed since the samples corresponds to the lists of chemicals developed by regulatory processes and held by regulatory agencies and an NGO. We collected information on hazardousness, economic parameters and scientific knowledge for all the chemicals to the extent to which the secondary data was available.

Data collection

Data was retrieved from online websites by Mikael Gustavsson (C&L/REACH/Lists) and Erik Kristiansson (PubMed).

All date information below as (YY-MM-DD)

The Classification and Labeling Inventory held by the European Chemicals Agency (ECHA).
Accessed originally 2019-10-15
Registration dossiers held by the European Chemicals Agency (ECHA).
Accessed originally 2019-07-11
PubMed held by the National Centre for Biotechnology information (NCBI)
Accessed originally 2020-04-06
The Candidate List of Substances of Very High Concern held by ECHA
Final version obtained 2020-02-25
The Authorization List held by ECHA.
Final version obtained 2020-02-25
The SIN (Substitute It Now) List developed and held by the International Chemical Secretariat (ChemSec).
Final version obtained 2020-02-25
The PRIO Phase out list developed and held by the Swedish Chemical Agency
Final version obtained 2020-02-25

Timing and spatial scale

Data collection started in July 2019 and ended in February 2020.

We sampled data on hazardousness, economic parameters and scientific knowledge at the start of the project and developed the assessment pipleine using the lists of chemicals downloaded at the onset of the project. Once the pipleine was developed a new set of lists were downloaded to ensure that the assessment of listed compounds was done with an up-to-date sample.

The spatial scale of the data corresponds to the area in which chemicals are regulated under the European union REACH regulation, that is the European Economic Area (EU+Island+Norway+Liechtenstein).

Data exclusions

Initial assessments were performed to remove parameters with high correlation as such parameters make it difficult to interpret the logit-model. The fit of all logit-models was determined using the adjusted McFadden pseudo R2, which penalizes model overfitting by accounting for the number of parameters included in the modeling. All collected data is present Zenodo (see Data Analysis section of the reporting card).

Reproducibility

Several robustness checks were implemented to verify the consistency of the results to alternative parameters. No physical experiments were conducted during the project. All code and data needed to reproduce the results as presented are available (see Data Analysis section of reporting card).

Randomization

Randomization is not relevant to our study since our regression analysis is based on all observation in our data. Moreover, our samples of chemicals include all chemicals registered under REACH and those included on exogenously determined lists of chemicals.

Blinding

Blinding is not relevant in our study as we only use data generated by external groups to perform the regression analysis. Also, all lists which are used in the data comparisons are developed by external groups (ECHA, ChemSec, KEMI)

Did the study involve field work? ☐ Yes ☒ No

Reporting for specific materials, systems and methods

We require information from authors about some types of materials, experimental systems and methods used in many studies. Here, indicate whether each material, system or method listed is relevant to your study. If you are not sure if a list item applies to your research, read the appropriate section before selecting a response.

Materials & experimental systems

n/a	Involved in the study
☒	☐ Antibodies
☒	☐ Eukaryotic cell lines
☒	☐ Palaeontology and archaeology
☒	☐ Animals and other organisms
☒	☐ Clinical data
☒	☐ Dual use research of concern

Methods

n/a	Involved in the study
☒	☐ ChIP-seq
☒	☐ Flow cytometry
☒	☐ MRI-based neuroimaging
